# A TCRVβ6 ^+^ Th1 cell subsets during *Salmonella enterica* serovar Typhimurium infection

**DOI:** 10.1111/jcmm.17862

**Published:** 2023-08-21

**Authors:** Yue Ai, Shao Wei, Jianwei Huang, Mengyao Wang, Yazhi Xue, Linli Wang, Hongbing Han

**Affiliations:** ^1^ Beijing Key Laboratory of Animal Genetic Improvement College of Animal Science and Technology China Agricultural University Beijing China; ^2^ Key Laboratory of Animal Genetics Breeding and Reproduction of the Ministry of Agriculture and Rural Affairs College of Animal Science and Technology China Agricultural University Beijing China; ^3^ Frontiers Science Center for Molecular Design Breeding(MOE) China Agricultural University Beijing China

To the Editor,

*Salmonella* infection causes morbidity and mortality worldwide, which is a substantial burden in developing and developed countries.^1^ Normally, activated T cells are indispensable for resistance to infection of *Salmonella*.^2^, ^3^ T cell antigen receptors (TCRs) specifically recognizing pathogen peptides presented by the major histocompatibility complex (MHC) is a key step during activating cell‐mediated adaptive immunity. Due to the enormous diversity of the TCR repertoire, the TCRs that specifically recognize the MHC complex (pMHC) of *S*. Typhimurium peptide were not fully uncovered. TCRs are composed of αβ polypeptide chain (~95%) or γδ polypeptide chain (~5%).^4^ αβ T cells were essential to resolve infection with *S*. Typhimurium, but not γδ T cells^3^ Each chain of TCRs consists of a variable region (V region), a joining region (J region), and a constant region (C region), of which β chain contains a diversity region (D region). Hypervariable complementarity‐determining regions (CDR3), the primary site of antigen‐specific recognition, are formed at the junction of different V(D)J segment rearrangements^5^, ^6^ (Figure 1A). Our study focused on the exploration of βTCRs with specific recognizing *S*. Typhimurium antigens.

**FIGURE 1:**
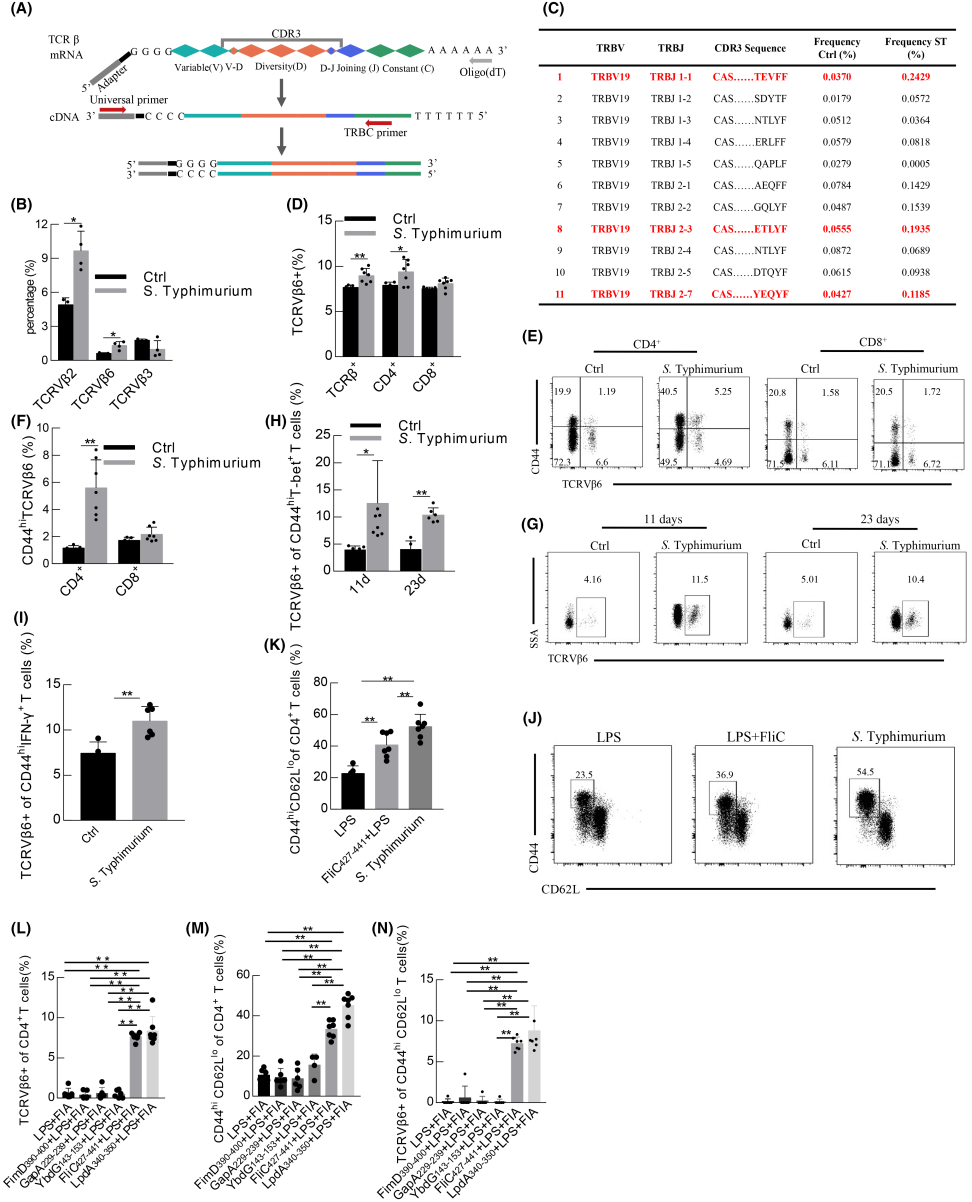
A TCRVβ6^+^ Th1 cell subsets during *Salmonella enterica* serovar Typhimurium infection. (A) A schematic diagram on βTCR repertoire of spleens in mice acquired by 5'RACE and PCR. (B) The frequency of different TRBV genes in infection or control (*n* = 3–4). (C) The frequency of CDR3 homologous sequences of TCRVβ6 in *S*. Typhimurium infection. (D) The proportion of TCRVβ6^+^ in total TCRβ^+^, CD4^+^ and CD8^+^ respectively in infection or control (*n* = 5–7). (E, F) The proportion of CD44^hi^ TCRVβ6^+^ cells in CD4^+^ and CD8^+^ T cells respectively at 11 days after infection. (G, H) The proportion of TCRVβ6^+^ CD44^hi^ T‐bet^+^ T cells in infection or control. (I) The percentage of TCRVβ6^+^ cells in CD44^hi^ IFN‐γ^+^ T cells in infection or control (*n* = 5–8). (J, K) The frequency of CD44^hi^ CD62L^lo^ cells in CD4^+^ T cells in mice injected with only LPS, FliC_427‐441_ peptide^+^ LPS, or infected *S*. Typhimurium (*n* = 5–7). L‐N. The percentage of TCRVβ6^+^ CD4^+^ T cells, CD44^hi^ CD62L^lo^ CD4^+^ T cells, and TCRVβ6^+^ CD44^hi^ CD62L^lo^ T cells in mice injected peptides 2 weeks after the last immunization (*n* = 6–7). Data were presented as mean ± SD, *p < 0.05, **p < 0.01.

Each T cell harbours a single TCR generally,^5^ therefore the expression level of the β chain represents the expansion degree of a single T cell clone. βTCR repertoire (the small DNA fragment 250–500 bp) in spleens of the C57BL/6J mice was acquired by 5'RACE and PCR amplification and was sequenced using the high throughput sequencing platform Miseq (Figure 1A, Figure S1 and Table S2). The sequences and structures of the β chain were analysed by the IMonitor tool.^4^ There was no significant difference in the number and average length of CDR3 between C57BL/6J mice infected and uninfected with *S*. Typhimurium(CVCC541). However, the clonotypes and Shannon Index of CDR3 were decreased in infected mice (Figure S2A–E). *S*. Typhimurium infection significantly increased the frequency of TCRVβ2 and TCRVβ6 (also called TCRβ variable, TRBV1 and TRBV19, respectively) and decreased the level of TCRVβ3 (TRBV26) (Figure S3 and Figure 1B). Because TCRVβ2 has been identified in mice during *S*. Typhimurium infection,^3^ we focused on the TCRVβ6. The abundance of CDR3 homologous sequences including paired TRBV19‐TRBJ1‐1, TRBV19‐TRBJ2‐3 and TRBV19‐TRBJ2‐7 was significantly increased in mice infected with *S*. Typhimurium (Figure 1C). Further, flow cytometry analysis proved that the proportion of TCRVβ6^+^ in total TCRβ^+^ was also accumulated (Figure 1D and Figure S4A). CD4^+^ T cells play multiple and essential roles during *Salmonella* infection^2^ In C57BL/6J mice infected with *S*. Typhimurium, the TCRVβ6^+^ CD4^+^ T cell clones were expanded, but not TCRVβ6^+^ CD8^+^ T cells (Figure 1D and Figure S4A). Meantime, activated TCRVβ6^+^ CD4^+^ T cells, CD44^hi^ TCRVβ6^+^ cells, were increased from 1.2% to 5.6% (Figure 1E,F). However, *S*. Typhimurium infection did not influence the expansion of TCRVβ3^+^ CD4^+^ and TCRVβ3^+^ CD8^+^ T cells (Figure S4B–E). In addition, in spleens and inguinal lymph nodes of the BALB/c mice that show more Th2‐skewed responses, CD44^hi^ TCRVβ6^+^ cells were also expanded at 11 days after *S*. Typhimurium infection (Figure S5A–D). These data suggested TCRVβ6^+^ CD4^+^ T cell clones were expanded and activated during *S*. Typhimurium infection.

Th1 cell subset expressing transcription factor T‐bet is responsible for resolving *Salmonella* infection through the production of IFN‐γ and TNF‐α.^7^ Our previous studies have found that Th1 cells were significantly activated at 11 days after *S*. Typhimurium infection to increase the clearance of *S*. Typhimurium.^8^ Therefore, we investigated the degree of the TCRVβ6^+^ T‐bet^+^ cells expansion. First, the proportion of CD44^hi^ T‐bet^+^ T cells in spleens was significantly increased at the 11 and 23 days after *S*. Typhimurium infection, while at the 23 days, it was lower than that 11 days (Figure S6A, B), which was consistent with that in inguinal lymph nodes (Figure S6C,D). Meanwhile, CD44^hi^ IFN‐γ^+^ CD4^+^and CD44^hi^ TNF‐α^+^ CD4^+^ T cells were activated (Figure S6E–F, G–H). Second, the proportion of CD44^hi^ TCRVβ6^+^ T‐bet^+^, CD44^hi^ TCRVβ6^+^ IFN‐γ^+^ and CD44^hi^ TCRVβ6^+^ TNF‐α^+^ T cells were significantly increased at 11 and 23 days after *S*. Typhimurium infection (Figure 1G–I; Figure S6I‐K), while CD44^hi^ TCRVβ3^+^ T‐bet^+^ cells had no obvious change (Figure S6L,M). These results indicated that the production of TCRVβ6^+^ Th1 cells was triggered during *S*. Typhimurium infection in C57BL/6J mice. Although the proportion of CD44^hi^ T‐bet^+^ T cells in spleens and inguinal lymph nodes were also significantly increased at 11 days after *S*. Typhimurium infection in BALB/c mice (Figure S7A–D), the proportion of CD44^hi^ TCRVβ6^+^ T‐bet^+^ cells was reduced in spleens and inguinal lymph nodes, which was contrary to C57BL/6J mice (Figure S7E–H). These results suggested that the expansion of CD44^hi^ TCRVβ6^+^ T‐bet^+^ cells was different in C57BL/6J and BALB/c mice with *S*. Typhimurium infection.

The percentage of CD44^hi^ T‐bet^+^ Th1 cells was decreased at 23 days after *S*. Typhimurium infection compared to 11 days (Figure S6A,B). The percentage of CD44^hi^ FOXP3^+^ (a marker of Treg cells) CD4^+^ T cells was significantly increased in the spleen and inguinal lymph nodes of C57BL/6J mice at 23 days after *S*. Typhimurium infection (Figure S8A–D). However, the frequency of CD44^hi^ TCRVβ6^+^ FOXP3^+^cells was almost the same in infected mice compared with control (Figure S8E,F). Furthermore, the percentage of TCRVβ3^+^ CD44^hi^ FOXP3^+^ cells was significantly decreased after *S*. Typhimurium infection (Figure S8G, H). Taken together, increased Treg cells might impair the production of Th1 cells at the late stage of *S*. Typhimurium infection (at 23 days), and neither TCRVβ6^+^ nor TCRVβ3^+^ Treg cells played a dominant role in the process of immunosuppression during *S*. Typhimurium infection.

Lastly, *S*. Typhimurium antigens activating TCRVβ6^+^ CD4^+^ T cells were identified. *S*. Typhimurium peptides FliC_427‐441_ could trigger CD4^+^ T cell response.^9^ Therefore, FliC_427‐441_ peptide was injected into mice with LPS, and the numbers of CD44^hi^ Ki67^+^ and CD44^hi^ CD62L^lo^ in CD4^+^ T cells were higher significantly than that in mice injected with only LPS, but lower than that in mice infected *S*. Typhimurium (Figure 1J, K and Figure S9A, B). It demonstrated that CD4^+^ T cells were expanded and activated by injecting the FliC_427‐441_ peptide. In addition, some other antigen peptides of *S*. Typhimurium could also activate CD4^+^ T cells. Thus, six peptides^9^ were selected to immunize C57BL/6J mice with LPS and FIA (incomplete adjuvant) (Table S1). The proportion of TCRVβ6^+^ CD4^+^ T cells was significantly increased in mice injected with FliC_427‐441_ or LpdA_340‐350_ (Figure 1L and Figure S9C). Simultaneously, activated CD4^+^ T, CD44^hi^ CD62L^lo^ and CD44^hi^ Ki67^+^ cells were accumulated (Figure 1M and Figure S9D–F). Further, the percentage of TCRVβ6^+^ T cells was higher significantly in activated CD4^+^ T cells (Figure 1N and Figure S9G–I). These data indicated the FliC_427‐441_ and LpdA_340‐350_ could induce the activation of TCRVβ6^+^ CD4^+^ T cells.

In conclusion, we identified that the TCRVβ6^+^ CD4^+^ T cell clones were expanded and activated in C57BL/6 and BALB/c mice during *S*. Typhimurium infection, and the TCRVβ6^+^ Th1 cell subset played a key role to resolve *S*. Typhimurium infection in C57BL/6 mice. Moreover, we confirmed *S*. Typhimurium antigen peptides, FliC_427‐441_ and LpdA_340‐350_ were essential for activating TCRVβ6^+^ CD4^+^ T cells.

## AUTHOR CONTRIBUTIONS

**Yue Ai:** Data curation (equal); formal analysis (equal); writing – original draft (equal). **Shao Wei:** Data curation (lead); formal analysis (lead); writing – original draft (lead). **Jianwei Huang:** Formal analysis (equal). **Mengyao Wang:** Writing – review and editing (equal). **Yazhi Xue:** Writing – original draft (equal). **Linli Wang:** Writing – review and editing (equal). **Hongbing Han:** Conceptualization (lead); funding acquisition (lead); project administration (lead); supervision (lead); writing – review and editing (lead).

## FUNDING INFORMATION

This study was supported by the National Key R&D Program of Intergovernmental Key Projects in China (2018YFE0101700).

## CONFLICT OF INTEREST STATEMENT

No potential conflict of interest was reported by the authors.

## Supporting information


Data S1

## Data Availability

Data sharing not applicable to this article as no datasets were generated or analysed during the current study.
