# Supplementary material for: A TCRVβ6 + Th1 cell subsets during Salmonella enterica serovar Typhimurium infection
Source: J Cell Mol Med. 2023 Aug 21;27(21):3414–7. doi: 10.1111/jcmm.17862 (PMC10623519; doi:10.1111/jcmm.17862)
Supplement: Supplementary file 1 — Data S1 [file JCMM-27-3414-s001.pdf]

Materials and methods

Ethics statement

All experimental animal protocols were approved by the Animal Care and Use Committee at China Agricultural University (Aw61111202-1-2).

Mice

Obtained from Beijing Vital River Laboratory Animal Technology Co., Ltd., female C57BL/6J mice (8 weeks) and female Balb/C mice (8 weeks) were kept under s pecific-pathogen-free conditions.

Bacterial strains

Salmonella enterica serovar Typhimurium CVCC541 strain was acquired from the Chinese Veterinary Culture Collection Center in the study and cultured in LB broth at 37°C. The bacterial dose was determined on LB agar plates at 37°C.

Synthetic peptides

Peptides were synthesized by GL Biochem (Shanghai) Ltd (Table S1). All peptides were dissolved in sterile water at a concentration of 2 mg/ml and further diluted in buffer for using in the various assays.

Infection and immunization

In infection experiment, after 2 weeks acclimatization, the mice were infected i ntravenously with *S*. Typhimurium strains CVCC541 suspended in 200μL of PBS (2×10^5, 1×10^5 or 1×10^4CFU), or injected only 200μL PBS for control groups. In immunization experiment, the mice were immunized intravenously with different 60 μg peptides plus 6μg LPS (Sigma) as adjuvant for 8 days or were injected subcutaneously with 100 μg of peptide in 10μg LPS and 100μL FIA in a total volume of 200 μl twice at a one-week interval, two weeks after the last immunization , mice spleens were removed for further analysis.

PCR amplification of CDR3β region

Spleens from mice were mashed in liquid nitrogen. Then total RNA was extracted by Trizol and reverse transcribed by SMARTer 5'RACE kit (Clontech, USA) as Fig. 1A. The PCR amplication was optimized as follows: partial long primer sequence provided

by 5'RACE kit was used as forward primer and constant β sequence as primer (Table S2). cDNA was amplified using KOD-Plus-Neo (TOYOBO, Japan) and the protocol was as follows: 2 min at 94°C; 20 cycles of 10s at 98°C, 30s at 60°C, and 20 s at 68°C, followed by final extension of 7 min at 68°C. In order to enrich CDR3 region, the first round amplication products were diluted and performed the second round of PCR with the same primers. About 250-500 bp fragment including CDR3 region was excised and purified by Gel Extraction with the NucleoSpin Gel and PCR Clean-Up Kit (Clontech, USA) (Fig. S1). Then libraries were prepared and sequenced by high throughput sequencing platform Miseq.

Determination of CDR3β sequences

The changes of CDR3 region, TRBV gene usage and TRBV-TRBJ gene pairing were analyzed by IMonitor software(1). The samples were sequenced on the Illumina Miseq platform. Raw resequencing reads were filtered according to chastity score and trimmed on the basis of quality score. Then the filtered sequencing data were processed and filtered using IMonitor. Clean sequences were aligned to V, D and J ref alleles (IMGT database, www.imgt.org), and V/J/D gene segments were assigned for each clone. The alignment parameter for V gene: -W 15 –K 3 –v 1 –b 3, the alignment parameter for D gene: -W 4 -K 3 -v 3 -b 5, the alignment parameter for J gene: -W 10 -K 3 -v 1 -b 3.

Analysis of TCRβ differences between groups

In this experimental study, the seven samples were divided into two groups: four samples in the infected group and three samples in the control group. The differences of TCR frequency between infected and control group were calculate by a house R script based on Mann-Whitney- U test. Significance was set at a $P<0.05$.

Flow cytometry

Spleens and inguinal lymph nodes from mice were mashed on 70 μ m cell strainer in PBS. 1×10^6 single cells were incubated with Zombie Aqua Fixable Viability Kit (Biolegend, USA) at room temperature, in the dark, for 15 min. Next, cells were resuspended in buffer (PBS containing 2% FBS) and blocked in Fc block (anti-mouse CD16/32, Clone S17011E, Biolegend, USA) at 4°C for 10 min. Cells were then incubated with following antibodies: anti-CD11b (clone M1/70, Biolegend, USA), anti-

F4/80 (clone BM8, Biolegend, USA), anti-CD19 (clone 6D5, Biolegend, USA), anti-CD11c (clone N418, Biolegend, USA), anti-CD3 (clone 17A2, Biolegend, USA), anti-TCRβ (H57-597, Biolegend, USA), anti-CD4(clone RM4-5, Biolegend, USA), anti-CD44 (clone IM7, Biolegend, USA,), anti-T-bet (Clone 4B10, Biolegend, USA), anti-IFN-γ (clone XMG1.2, Biolegend, USA) , anti-TNF-α (clone MP6-XT22, Biolegend, USA), anti-TCRVβ6 (clone RR4-7, Biolegend, USA), anti-TCRVβ3 (clone KJ25, BD,USA), anti-CD62L (clone MEL-14, Thermo, USA), anti-FOXP3 (Clone FJK-16s, Thermo, USA), anti-Ki67 (clone SolA15, Thermo, USA). The cell surface antibodies were incubated for 20 min at 4 °C in the dark. Intracellular Ki67, T-bet and FOXP3 were detected using Foxp3/Transcription Factor Staining Buffer Set (eBioscience, USA) according to manufacturers' instructions. For the detection of IFN-γ and TNF-α, 2×106 spleen cells were incubated in RPMI 1640 supplemented with 10% FBS plus Leukocyte Activation Cocktai at 37°C for 5 h. The levels of IFN-γ and TNF-α were estimated with Foxp3/Transcription Factor Staining Buffer Set as above.

Statistical analysis

Data were presented as mean ± SEM. The differences were analyzed by independent-samples t test or one-way ANOVA with GraphPad Prism.v5.0 and SPSS. The statistical significance was defined at $p < 0.05$.

Table S1: The sequences of peptides.

	Peptides	sequences
1	$YbdG_{143\text{-}153}$	VFKDPILGLVA
2	$FimD_{390\text{-}400}$	IAGLPAGLTAY
3	$GapA_{229\text{-}239}$	AFRVPTPNVSV
4	$FljB_{391\text{-}401}$	IDGKTYNASKAAGHDF
5	$FliC_{427\text{-}441}$	VQNRFNSAITNLGNT
6	$LpdA_{340\text{-}350}$	AGKKHYFDPKVIPSIAY

Table S2: the second PCR amplification primers

Upstream primers	5’AAGCAGTGGTATCAACGCAGAGT3’
Downstream primers	5’ ACCTTGGGTGGAGTCACATTTC 3’

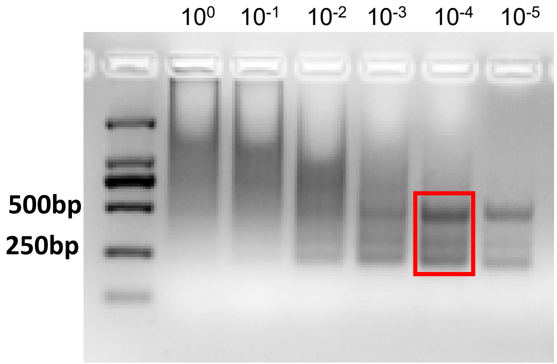

Fig. S1 PCR amplification of CDR3β region, Gel electrophoresis of the 250-500 bp fragment including CDR3 region.

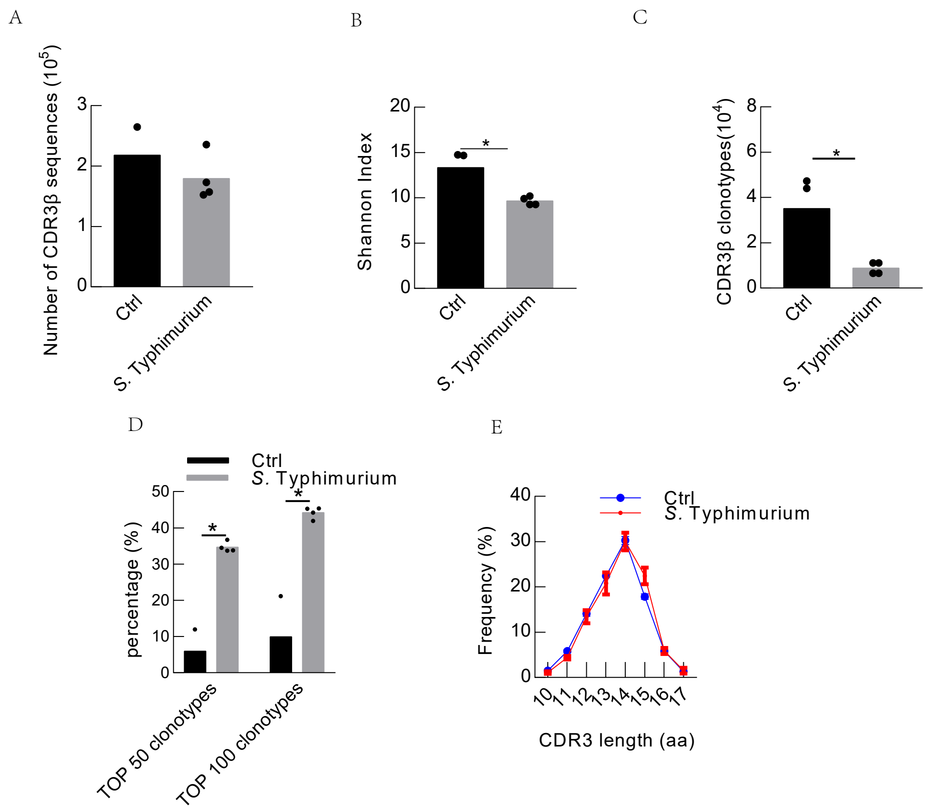

Fig. S2 (A) the number of CDR3β sequences. (B) Shannon Index of CDR3β sequences. (C) clonotypes of CDR3β sequences. (D) the percentage of top 50 and top 100 CDR3β clonotypes. (E) the average length of CDR3 in mice infected (2×10^5CFU,11days) or uninfected with *S.* Typhimurium (CVCC541). (n=3-4), $^*p<0.05$.

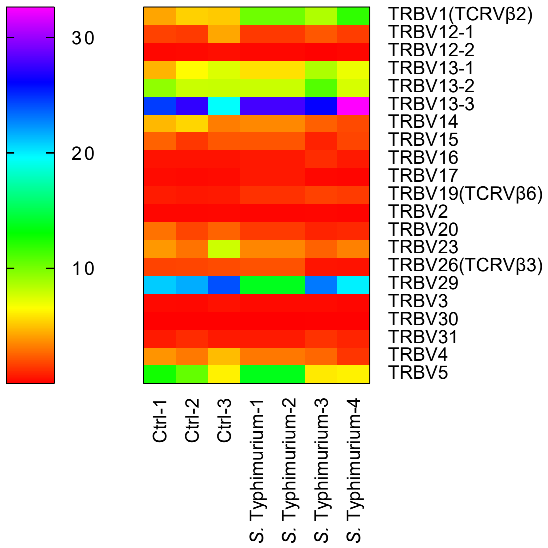

Fig. S3 Heat Map showing the frequency of different TRBV genes segments in mice infected (2×10^5CFU,11 days) or uninfected with *S.* Typhimurium (n=3-4).

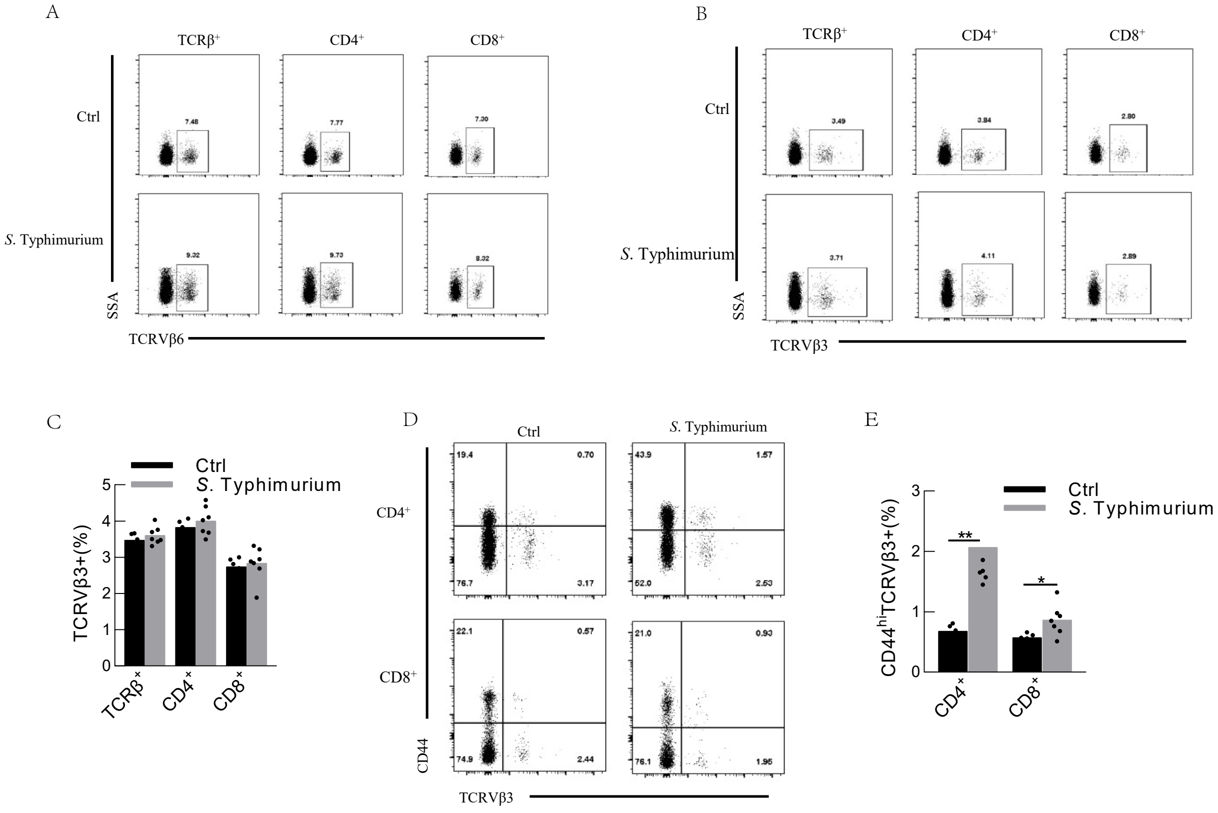

Fig. S4 (A) Flow cytometry analyzed the proportion of TCRVβ6$^+$ in total TCRβ$^+$, CD4$^+$ and CD8$^+$ T cells respectively in mice infected (2×10^{5}CFU,11days) or uninfected with *S.* Typhimurium. (B)-(C) Flow cytometry and statistical analysis the proportion of TCRVβ3$^+$ cells in TCRβ$^+$, CD4$^+$ and CD8$^+$ T cells in mice infected (2×10^{5}CFU,11days) or uninfected with *S.* Typhimurium. (D)-(E) Flow cytometry and statistical analysis the proportion of CD44^{hi} TCRVβ3$^+$ cells in CD4$^+$ and CD8$^+$ T cells respectively in mice infected (2×10^{5}CFU,11days) or uninfected with *S.* Typhimurium. (n=5-7), *$p<0.05$, **$p<0.01$.

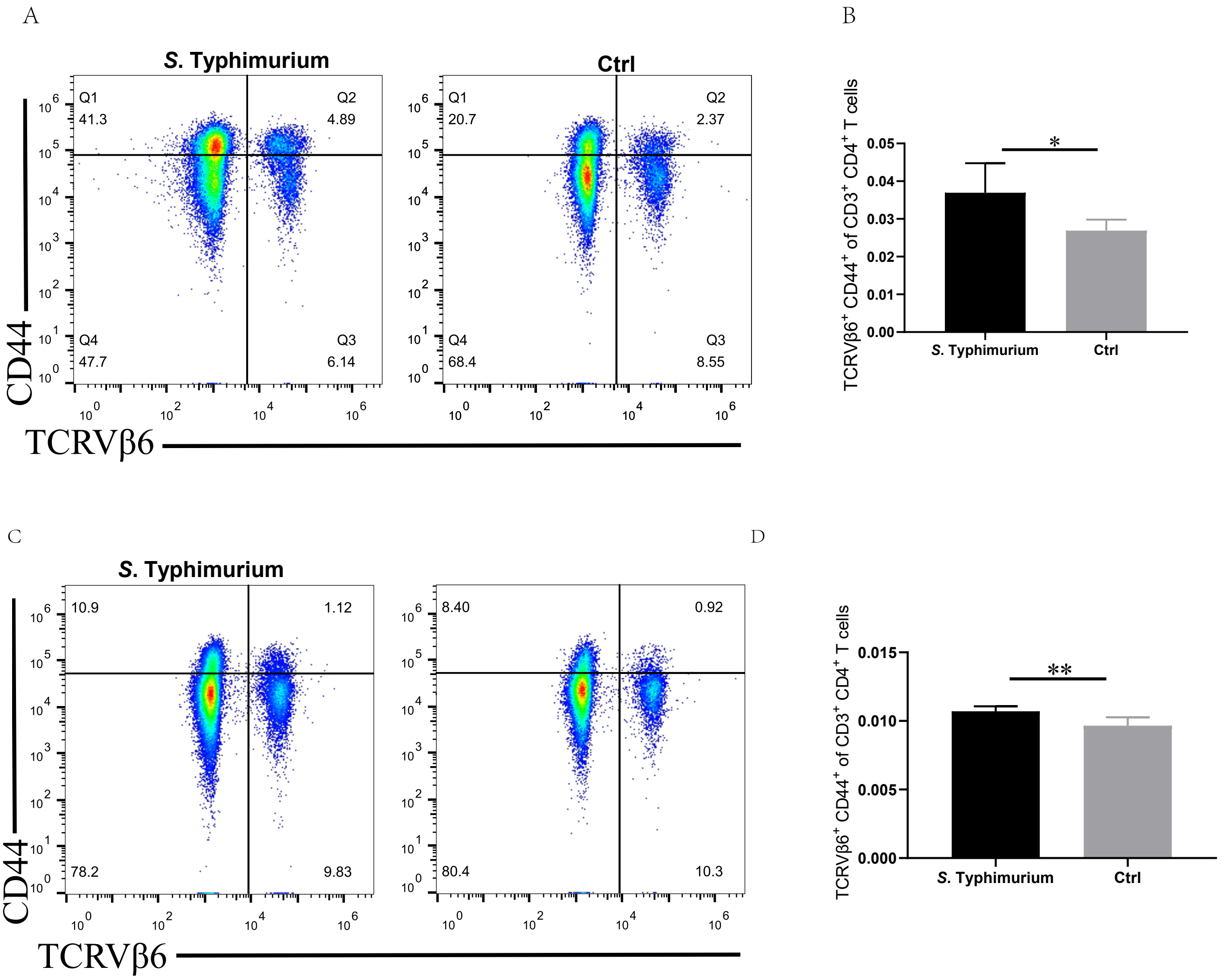

Fig. S5 (A)-(B) Flow cytometry and statistical analysis the proportion of CD44^{hi} TCRVβ6^{+} cells in CD3^{+} CD4^{+} T cells respectively in spleens of Balb/C mice infected (1×10^{4}CFU,11days) or uninfected with *S.* Typhimurium. (C)-(D) Flow cytometry and statistical analysis the proportion of CD44^{hi} TCRVβ6^{+} cells in CD3^{+} CD4^{+} T cells respectively in inguinal lymph nodes of Balb/C mice infected (1×10^{4}CFU,11days) or uninfected with *S.* Typhimurium. (n=6), *$p<0.05$, **$p<0.01$.

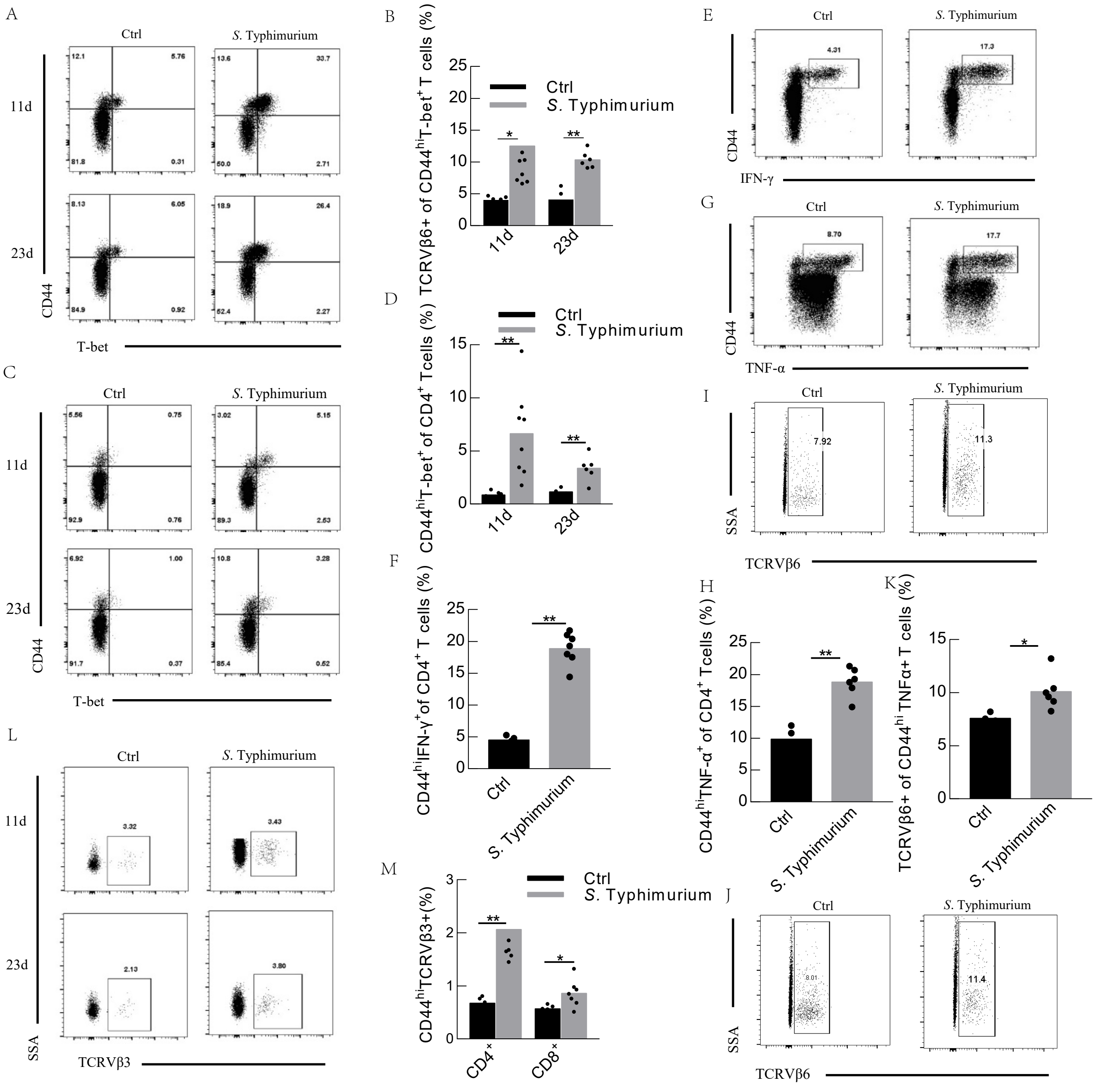

Fig. S6 (A)-(B) Flow cytometry and statistical analysis the proportion of CD44^{hi} T-bet^{+} T cells in CD4^{+} T cells of spleens (1×10^{5}CFU,11 or 23 days). (C)-(D) Flow cytometry and statistical analysis the proportion of CD44^{hi} T-bet^{+} T cells in CD4^{+} T cells of inguinal lymph nodes (1×10^{5}CFU,11 or 23 days). (E)-(F) Flow cytometry and statistical analysis the proportion of CD44^{hi} IFN-γ^{+} cells in CD4^{+} T cells of spleens (1×10^{5}CFU, 11 or 23 days). (G)-(H) Flow cytometry and statistical analysis the proportion of CD44^{hi} TNF-α^{+} cells in CD4^{+} T cells of spleens (1×10^{5}CFU, 11 or 23 days). (I) Flow cytometry analysis the percentage of TCRVβ6^{+} cells in CD44^{hi} IFN-γ^{+} T cells of spleens (1×10^{5}CFU, 11 or 23 days). (J)-(K) Flow cytometry and statistical analysis the percentage of TCRVβ6^{+} cells in CD44^{hi} TNF-α^{+} T cells of spleens (1×10^{5}CFU, 11 or 23 days). (L)-(M) Flow cytometry and statistical analysis the proportion of TCRVβ3^{+} cells in CD44^{hi} T-bet^{+} T cells (1×10^{5}CFU, 11 or 23 days). (n=5-8), *$p<0.05$, **$p<0.01$.

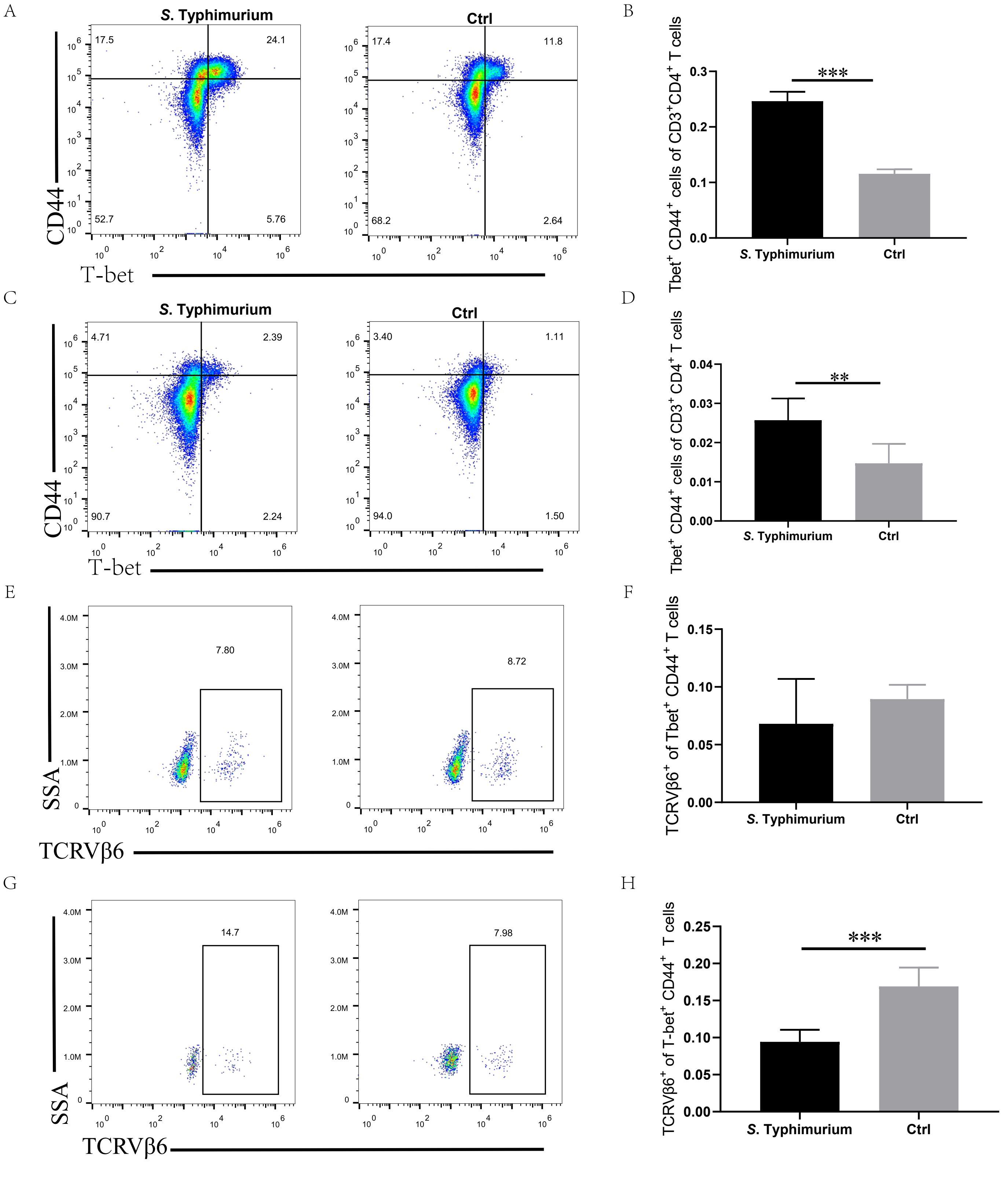

Fig. S7 (A)-(B) Flow cytometry and statistical analysis the proportion of CD44^{hi} T-bet^{+}T cells in CD3^{+} CD4^{+} T cells in spleens of Balb/C mice infected (1×10^{4}CFU, 11days) or uninfected with *S.* Typhimurium. (C)-(D) Flow cytometry and statistical analysis the proportion of CD44^{hi} T-bet^{+} T cells in CD3^{+} CD4^{+} T cells in inguinal lymph nodes of Balb/C mice infected (1×10^{4}CFU,11days) or uninfected with *S.* Typhimurium. (E)-(F) Flow cytometry and statistical analysis the proportion of TCRVβ6^{+} in CD44^{hi} T-bet^{+} T cells in spleens of Balb/C mice. (G)-(H) Flow cytometry and statistical analysis the proportion of TCRVβ6^{+} in CD44^{hi} T-bet^{+} T cells in inguinal lymph nodes of Balb/C mice. (n=6), *$p<0.05$, **$p<0.01$.

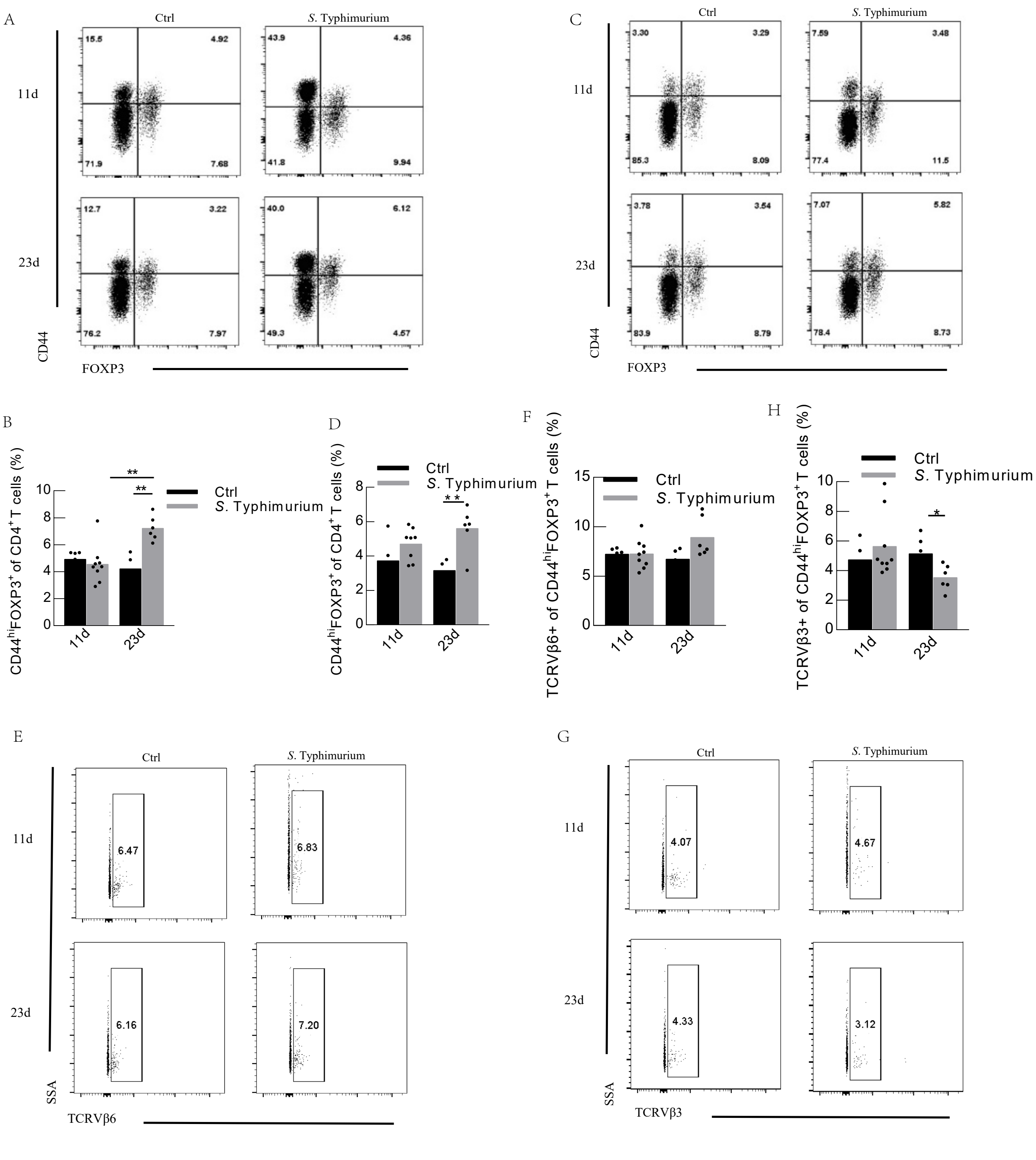

Fig. S8 (A)-(B) Flow cytometry and statistical analysis the frequency of CD44^{hi} FOXP3^{+} in CD4^{+} T cells of spleens at 11 and 23 days after 1×10^{5} CFU *S.* Typhimurium infection. (C)-(D) Flow cytometry and statistical analysis the frequency of CD44^{hi} FOXP3^{+} in CD4^{+} T cells of inguinal lymph nodes at 11 and 23 days after 1×10^{5} CFU *S.* Typhimurium infection. (E)-(F) Flow cytometry and statistical analysis the frequency of the TCRVβ6^{+} cells in CD44^{hi} T-bet^{+} T cells of spleens at 11 and 23 days after 1×10^{5} CFU *S.* Typhimurium infection. (G)-(H) Flow cytometry and statistical analysis the frequency of TCRVβ3^{+} cells in CD44 ^{hi} T-bet^{+} T cells of spleens at 11 and 23 days after 1×10^{5} CFU *S.* Typhimurium infection. (n=6-9), $^{*}p< 0.05$, $^{**}p<0.01$.

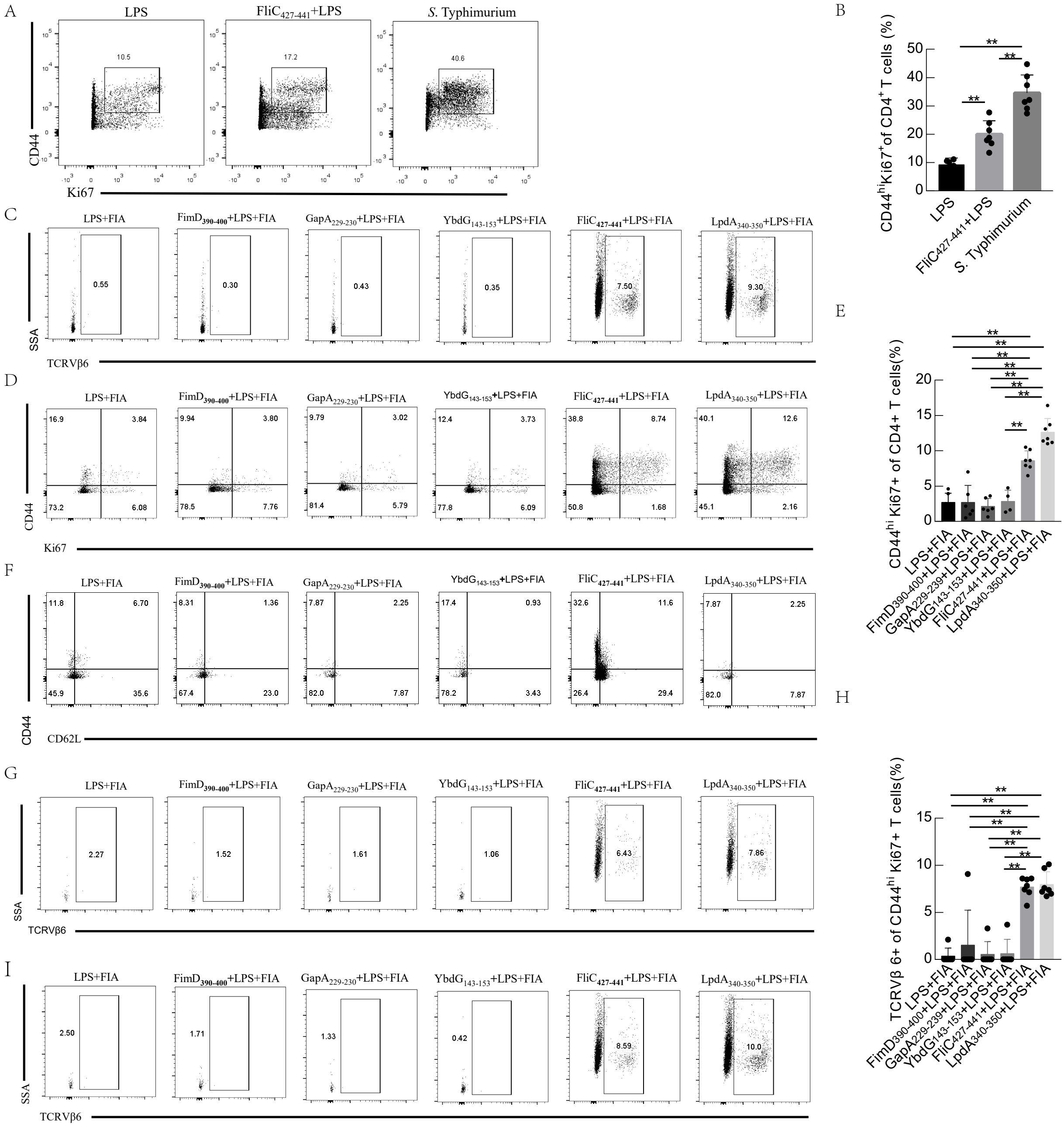

Fig. S9 (A)-(B) Flow cytometry and statistical analysis the frequency of CD44^{hi} Ki67^{+} cells in spleens CD4^{+} T cells of mice injected with only LPS, FliC$_{427-441}$ peptide and LPS and infected *S.* Typhimurium (1×10^{5} CFU, 8 days) (n=5-7). (C)Flow cytometry the frequency of TCRVβ6^{+} cells in spleens CD4^{+} T cells of mice injected with six peptides respectively two weeks after the last immunization (n=6-7). (D)-(E) Flow cytometry and statistical analysis the frequency of CD44^{hi} Ki67^{+} cells in spleens CD4^{+} T cells of mice injected with six peptides respectively two weeks after the last immunization (n=6-7). (F)-(G) Flow cytometry and statistical analysis the frequency of TCRVβ6^{+} cells in CD44^{hi} Ki67^{+} T cells of mice injected with six peptides respectively two weeks after the last immunization (n=6-7). (H) Flow cytometry analysis of the frequency of CD44^{hi} CD62L^{lo} cells in spleens CD4^{+} T cells of mice injected with six peptides respectively two weeks after the last immunization (n=6-7). (I) Flow cytometry analysis of the frequency of TCRVβ6^{+} cells in CD44^{hi} CD62L^{lo} T cells of mice injected with six peptides respectively two weeks after the last immunization (n=6-7). **$p<0.01$.

REFERENCES

1.Zhang W, Du Y, Su Z, Wang C, Zeng X, Zhang R, et al. IMonitor: A Robust Pipeline for TCR and BCR Repertoire Analysis. Genetics. 2015;201(2):459-72.
